# Retrospective Analysis of Complications Associated with Laparoscopic Cholecystectomy for Symptomatic Gallstones

**DOI:** 10.7759/cureus.5152

**Published:** 2019-07-16

**Authors:** FNU Amreek, Syed Zohaib Maroof Hussain, Munawar H Mnagi, Amber Rizwan

**Affiliations:** 1 General Surgery, New York University Langone Medical Center, New York, USA; 2 Surgery, Aga Khan University Hospital, Karachi, PAK; 3 General Surgery, Shaheed Mohtarma Benazir Bhutto Medical College Lyari, Karachi, PAK; 4 Family Medicine, Dr. Ruth Pfau Hospital, Karachi, PAK

**Keywords:** laparoscopic cholecystectomy, gallstones, conversion to open, retrospective, pakistan, complication rate, mortality rate

## Abstract

Introduction

Gallstones are the major cause of global morbidity. Laparoscopic approach has well-established advantages as compared to the conventional open procedure. It promises better recovery, lower morbidity, and lower postoperative pain, shortens the duration of hospital stay, and has a lower mortality rate. The aim of this study is to assess the frequency of complications in laparoscopic cholecystectomies indicated for symptomatic gallstones and also evaluate the rate of conversion.

Methods

In this retrospective analysis, all records of laparoscopic cholecystectomy, in patients of age ≥18 years, for symptomatic gallstones, from January 2015 till December 2018 in one of the largest public tertiary care hospitals in Pakistan were included.

Results

The rate of complications associated with laparoscopic cholecystectomy was 6.8%. Older age, obesity, and multiple pre-operative risk factors were associated with complications. The most common intra-operative complication was hemorrhage (1.3%) and most common postoperative complication was surgical site infection (2.7%). Our conversion rate was 3.6%. Both intra-operative and postoperative complications were more common in procedures which were converted to open.

Conclusion

The rate of complication and conversion to open in laparoscopic cholecystectomy is not very high. Older age, obesity, and multi-morbidity was associated with complications. Complicated procedures were more commonly needed to be converted to open.

## Introduction

Gallstones disease has been a major cause of global morbidity with varying degree of prevalence based on the geographical, racial, and ethnic parameters. Gallstones are at least three to four times more common in females. The incidence of gallstones increases with advancing age and are rare before 20 years of age [1]. In the United States (US), 15% of the adult population has gall bladder stones [2]. In Pakistan, the prevalence of gallstones has been reported to be 10% [3]. Higher incidence may be explained by the obesity epidemic and sedentary lifestyles [2].

Laparoscopic cholecystectomy (LC) was first introduced in the mid-1980s for symptomatic gallstones. Currently, it is the gold standard treatment for symptomatic gallstones [4]. In the initial decades, laparoscopic procedures were only restricted for well-experienced senior surgeons, however, studies gradually showed how the level of surgical expertise and the duration of practice did not impact the patient outcome in laparoscopic procedures. LC is a safe procedure commonly performed by surgical residents under the supervision of there attending [5]. Good procedure outcome and the reduced in-hospital stay have aided the dramatic shift from open to laparoscopic approach to gallstones. More than 90% of the 830,000 cholecystectomy in the US are performed using a laparoscopic approach [6].

Advantages of the laparoscopic approach include better recovery, lower morbidity, and lower postoperative pain, shortened duration of hospital stay, and lower mortality rate. However, intraoperative bleeding was seen to be unaffected by the selection of operative technique [7]. The incidence of major complications with LC remains as low as 5% [8]. There may be several factors contributing to the risk of conversion to an open procedure. Distorted anatomy, excessive bleeding, visceral injury, adhesion, equipment failure and surgeons expertise are all factors that increase conversion from LC to open approach [9]. The frequency of conversion has been reported to be 5% [10]. The aim of this study is to assess the frequency of complications in LC indicated for symptomatic gallstones and rates of conversion from laparoscopic to conventional open cholecystectomy.

## Materials and methods

In this retrospective analysis, all patient records of laparoscopic cholecystectomy from January 2015 till December 2018 were included. The study was conducted in one of the largest public tertiary care hospitals in Sind, Pakistan. The study was approved by the institutional review board.

All records were of patients of age ≥18 years of both genders. All records were of patients who were admitted from the outpatient department with symptomatic gallstones. Other conditions such as choledocholithiasis or dilated common bile duct (CBD >8 mm in diameter), acute cholecystitis, malignancy or mass at porta hepatis, and history of jaundice and/or previous abdominal or pelvic surgery were excluded from the analysis.

All data was entered and analyzed using SPSS for Windows version 23.0 (IBM Corp., NY, USA). Mean and the standard deviation was calculated for continuous variables. Frequencies and percentages were calculated for continuous variables. The correlation analysis was done for characteristics of patients with and without complications. Complications were compared for patients with complete laparoscopic procedures and in those open procedures were done. Chi-square was applied. P-value ≤0.05 was taken as significant.

## Results

During the three year period, 855 laparoscopic cholecystectomies were performed. There were 19 (2.2%) records which were incomplete such that neither any complication was mentioned nor “no intra/postoperative complication” was mentioned. In 778 (90.9%) records, “no intra/postoperative complication” was mentioned. Procedure-related intra/postoperative complication occurred in 58 (6.8%) patients. All records were included for analysis.

There were 312 males (36.5%) and 543 females (63.5%) in the sample. Their mean age was 48 ± 11 years. There were 487 (56.9%) patients of age less than 50 years and 368 (43.1%) were older than 50 years of age. Their mean body mass index (BMI) was 29.2 ± 4.5 kg/m^2^. There were 429 (50.2%) patients with BMI ≤ 29.9kg/m^2^ and 426 (49.8%) patients were obese with BMI more than 29.9kg/m^2^. All procedures were elective and no emergency procedure was performed. There were 353 (41.3%) patients identified as multi-morbid (≥2 chronic illnesses). The most common comorbidities were related to the cardiovascular system (n=211; 59.7%), followed by metabolic (n=179; 50.7%), and renal system (n=114; 32.3%).

Procedure-related complications were reported in 58 (6.8%) patients. The frequency of occurrence of different complications is shown in table 1. The rate of mortality was 0.2%.

**Table 1:** Frequency of occurrence of different complications in patients undergoing laparoscopic cholecystectomy (n=855) CBD - Common Bile Duct

Complications	Frequency (%)
No Complications	778 (90.9%)
Missing data	19 (2.2%)
Complications	58 (6.8%)
Intraoperative complications	
Intraoperative bleeding	11 (1.3%)
Bile duct injury	9 (1.1%)
Ligation of CBD	4 (0.5%)
Duodenal perforation	7 (0.8%)
Conversion to open procedure	31 (3.6%)
Postoperative complications	
Surgical site infection	23 (2.7%)
Jaundice	11 (1.3%)
Biliary peritonitis	10 (1.2%)
Intra-abdominal collections	6 (0.7%)
Fecal peritonitis	5 (0.6%)
Bile leakage	4 (0.5%)
Death	2 (0.2%)
Retained CBD stones	1 (0.1%)

Patient demographic profiles were then compared with the incidence of complications. Age, multi-morbidity, and obesity were significantly correlated with the incidence of complications as shown in table 2.

**Table 2:** Comparison of demographic characteristics of patients with and without procedure-related complications (n=855)

Variable	No complication n=797 (93.2%)	Complications n=58 (6.8%)	P-value
Age			
Less than 50 years	463 (58.1%)	24 (41.4%)	0.01
More than 50 years	334 (41.9%)	34 (58.6%)	
Gender			
Male	289 (36.3%)	23 (39.7%)	0.26
Female	508 (63.7%)	35 (60.3%)	
Multi-morbid patient			
Yes	315 (39.5%)	38 (65.5%)	0.0001
No	482 (60.5%)	20 (34.5%)	
Body mass index			
Less than 29.9 kg/m^2^	411 (51.5%)	18 (31.0%)	0.002
More than 29.9 kg/m^2^	386 (48.4%)	40 (69.0%)	

In 31 (3.6%) patients, laparoscopic procedure was converted to open laparotomy. In the remaining 824 (96.4%) patients, procedures were completed laparoscopically. The incidence of intra/postoperative complications were compared for both these groups. All of the intra/postoperative complications other than intra-abdominal collections and retained common bile duct stones were statistically more significant in patients who were converted to open procedures. All of the patients who developed duodenal perforation were opened for exploratory laparotomy. The differences are shown in table 3. 

**Table 3:** Comparison of intraoperative and postoperative complications in patients with successful laparoscopic procedure vs. converted to open procedure (n=855) CBD - Common Bile Duct

Complications	Laparoscopy Completed n=824 (96.4%)	Converted to open n=31 (3.6%)	P-value
Intraoperative complications			
Intraoperative bleeding	4 (0.4%)	7 (22.5%)	<0.0001
Bile duct injury	2 (0.2%)	7 (22.5%)	<0.0001
Ligation of CBD	3 (0.3%)	1 (3.2%)	0.012
Duodenal perforation	0 (0%)	7 (22.5%)	<0.0001
Postoperative complications			
Intra-abdominal collections	6 (0.6%)	0 (0%)	0.66
Bile leakage	3 (0.3%)	1 (3.2%)	0.012
Surgical site infection	20 (2.4%)	3 (9.6%)	0.014
Biliary peritonitis	8 (0.8%)	2 (6.4%)	0.002
Fecal peritonitis	4 (0.4%)	1 (3.2%)	0.030
Retained CBD stones	1 (0.1%)	0 (0%)	0.86
Jaundice	9 (0.9%)	2 (6.4%)	0.004
Death	0 (0%)	2 (6.4%)	<0.0001

## Discussion

Laparoscopic cholecystectomy has made its place as the gold standard procedure for management for gallstones. In comparison to the open procedure, it is being readily practised as a “day-surgery” procedure. However, like all other medical procedures, LC also has its complications. Some complications, which may occur due to surgeon or equipment factors, may be avoidable; however, others which are due to patient factor may be unavoidable. In this study, complications were more common in males, older patients, high-risk, and in obese patients. This study retrospectively analyzed that the rate of complications in patients undergoing elective LC for gallstones is 6.8% and only 3.6% procedures were converted to open.

This is a substantial study with a large sample and detailed analysis of intraoperative and postoperative complications. However, the study has its limitations too. The study design was retrospective and data was gathered from patient records, hence subjected to bias. Also, the differences in the duration of hospital stay between the two groups (open vs LC) could also not be included in the study due to missing data.

The need to convert a laparoscopic procedure into an open one should never be taken as a surgical failure. Instead, it is an attempt to minimize the risk of morbidity and mortality. With frequent use of LC, the rate of conversion has been dropping. This is because with time surgeons are gaining more experience in this minimally invasive technique. The literature reports the rate of conversion as 1-24% [11-14].

In the recent literature, complications associated with LC included massive hemorrhage (0.3-1%), bile duct injury (0.13%), duodenal injury (0.13%), colon injury (0.06%), and 9.8% patients needed conversion to open procedure [15]. Similarly, in a retrospective analysis of 10-year records, successful LC was performed in 94% patients, the postoperative morbidity rate was 1.5%, and conversion to open laparotomy occurred in 3.5% patients [16]. In another Saudi study, the rate of complications associated with LC was 7.5% with hemorrhage (35%) and infection (22.5%) being the most common [17]. In a nationwide large scale study, the complication rate with LC was 6.8%. Patient factors - age (>65 years), male gender, and higher comorbidity score - were associated with complications more frequently than surgeon or equipment related factors. Surgeon or hospital volume did not independently increase the risk of complications in their analysis [18]. In another study that compared the outcome of LC done by resident trainees and experienced surgeons, it was deduced that trainees could perform difficult LCs under careful supervision and it did not increase the risk of complications; however, trainees took a long time to complete the procedure. They reported a conversion rate of only 1.7% in their study [19]. In comparison, Indian study reported high complication rate (14%), bile leakage (35.3%) and subhepatic abscess (26.5%) as common complications as compared to hemorrhage (11.8%) [20] reported in most of the literature [15,16]. However, they did not mention the reasons behind high complication rate.

The outcome of LC majorly depends on patient factors and also surgeon and equipment factors. Like any other medical intervention, LC also has its complications. With a careful patient selection, timely medical intervention, and controlling surgeon and technical factors, LC has the potential to produce excellent results. It is the gold standard therapy for managing symptomatic gallstones. 

## Conclusions

The rate of complications associated with laparoscopic cholecystectomy and the rate of conversion to open laparotomy is low in this retrospective analysis conducted with patients of symptomatic gallstones. Older age, obesity, and multi-morbidity was associated with complications. Both intraoperative and postoperative complications were more common in procedures which were converted to open.
